# Medical and Physical Intelligence

**Published:** 1821-12

**Authors:** 


### Medical and Physical Intelligence.

ROYAL *College of Physicians.*—At the annual election of officers of the College, last month, Sir Henry Halford, Bart. was re-elected President, Dr. Currey, Treasurer, and Dr. Hue, Registrar; Dr. Frampton, Dr. Hume, Dr. Badham, and Dr. Lloyd were elected Censors; Sir Henry Halford, Bart., Dr. Hervey, Dr. Gower, Dr. Hue, and Dr. Bright, Commissioners under the Mad-House Act; and Dr. Powell, Secretary to the Commissioners.

*Zoology.*—Dr. Paolo Savi, assistant to the Professor of Botany in the University of Pisa, has discovered in different places in the Tuscan Apennines, a new species of salamander, remarkable for its figure and colours. He has named it *Salamandra perspicillata quinque palmis plantisque tetradactylis.* It has a spot on the top of the head in shape like a pair of spectacles, and also four toes on each foot, instead of three, like the common salamander.

*Hydrophobia.*—At Pavia, new trials have been made which prove the efficacy of *oxygenated muriatic acid* in subduing the hydrophobia. Dr. Previsali had prescribed it with success where the symptoms were advanced, in a liquid form, from a drachm to a drachm and a half daily, in citron water or syrup of citron.

*Milk.*—It is ascertained that morning's milk commonly yields some hundredths more cream than the evening's at the same temperature. That milked at noon furnishes the least. It would therefore be of advantage, in making butter and cheese, to employ the morning's milk, and to keep the evening's for domestic use. In milking cows this singular phenomenon is observed, that the milk obtained from one and the same milking differs considerably in quality; that, contrary to what might be expected, the first drawn is not the best, but that which is obtained last contains invariably the largest portion of cream. Every regular dairy-man knows that the last-drawn milk called *strippings,* is by far the richest, and that there is a gradation of fulness, from the first milking to the last.

*Air Pump Bath.*—Mr. David Brownrigg, Deputy Inspector of Hospitals, has after much trouble constructed a machine by which the whole body, except the head, may be subjected to the effects of the air pump bath, with or without medicated fumigation. He has tried its powers in a few cases, and has great reason to be satisfied with its medicinal agency, and will, as soon as his leisure admits, submit to the public a detached account of its construction and application.

### LIVERPOOL EYE INFIRMARY.

*Liverpool; Oct.* 25, 1821.

SIR, —The interest you take in promoting every department of medicine induces me to take the liberty of sending you the first Report of the "Liverpool Institution for curing Diseases of the Eye." Should any Cases worthy of insertion in the Medical and Physical Journal occur, I shall forward them to you. Meantime,

I have the honor to be, sir, your very obedient servant,

*To the Editor, &c.* ALEX. HANNAY, M.D.

---

*Medical Officers of the Institution.*

Dr. HANNAY, Physician.
Mr. BRANDRETH and Mr. LOFTUS, Consulting Surgeons.
Mr. BRACKENBURY, Operating Surgeon and Surgeon in Ordinary.

---

### MEDICAL REPORT.

*Patients admitted, from the* 1*st of July*, 1820, *to the* 1*st of July*, 1821.

| | |
|---|---|
| Acute Ophthalmia | 60 |
| Purulent Ophthalmia—Adults | 40 |
| Ditto Infants | 75 |
| Ophthalmia, with Pustules | 39 |
| Ditto, with Slough or Ulcer | 47 |
| Ditto, with Hypopion | 5 |
| Strumous Ophthalmia | 41 |
| Chronic Ophthalmia | 77 |
| Ditto, with granular Conjunctiva and vascular Cornea | 10 |
| Excrescence of the Conjunctiva | 1 |
| Pterigium | 3 |
| Opacities of the Cornea | 61 |
| Protrusions of the Iris | 5 |
| Inflammation of the Iris | 20 |
| Unnatural adhesions of the Iris, with disfigured, contracted, or closed Pupil | 25 |
| Staphyloma | 5 |
| Cataract | 41 |
| Amaurosis, organic and functional | 44 |
| Ditto, combined with Cataract | 6 |
| Paralysis of the Upper Eyelids | 3 |
| Tinea | 87 |
| Lippitudo | 13 |
| Inversion of the Eyelids | 2 |
| Eversion of the Eyelids | 1 |
| Tumors in the Eyelids | 7 |
| Hordeolum | 3 |
| Diseases of the Lachrymal Passages | 10 |
| Wounds and Injuries of the Eye | 8 |
| Malignant fungus of the Eyeball (fatal) | 1 |
| Total | 758 |

| | | |
|---|---|---|
| Out of which number .... | 528 | have been discharged, cured. |
| | 60 | ditto, more or less relieved. |
| | 20 | incurable or irregular. |
| Remaining upon the books | 150 | |

Eleven cases have been operated upon for Cataract; seven were restored to sight; two are now under absorption, and two have been unsuccessful on account of supervening inflammation. A share of the misfortune which followed the two last-mentioned operations may, with justice, be attributed to want of proper accommodation for the patients, and their neglect to call in assistance at the onset of inflammation.

---

### WINCHESTER EYE INFIRMARY.

*Winchester; Nov. 9th,* 1821.

Sir,

I observed in your Journal for October, an account of an Institution for the cure of Injuries and Diseases of the Eyes, which is attempted to be established in this city, and also some extracts from a letter of mine in defence of the County Hospital; but as these extracts are rather unconnected, I have taken the liberty of enclosing to you a copy of the circular address of Mr. H. G. Lyford and Mr. W. J. Wickham, who constitute the "small number of medical practitioners setting the plan on foot," together with a copy of my letter to the Governors of the County Hospital, which was meant as a reply to the said address. The object of my letter was to support my own credit and that of the hospital, both of which were by many of my friends considered to be indirectly attacked by the statement of my colleagues, and to propose an addition to our establishment, which must clearly obviate any necessity for a distinct Institution upon the ground of *public benefit.* This proposal, I am happy to inform you, the committee have unanimously resolved to accede to, and I trust you will also be happy to find, notwithstanding the glaring contrast between the statement in my letter and the report of the new Institution in the Hampshire Chronicle, that in the same space of time we have had treble the number of severe ophthalmic cases under treatment as in-patients at the Hospital, to what there has been at the Institution, which at most has never had more than one or two in at a time, and doubtless, the fifty others might also have been relieved at the Hospital, if the same pains had been taken to collect them from the country round: but, unless we can conceive our neighbouring practitioners to be themselves afflicted with the most inveterate opacities, these might safely have been spared an irksome pilgrimage for the purpose of swelling the number of votaries at this recently erected shrine of the New Light.—I have the honor to be, sir,

Your obedient servant,

*To the Editor, &c.*

Charles Mayo,
*Senior Surgeon to the County Hospital.*

[*Circular.*] *Winchester; July 6,* 1821.

Sir,—It is proposed to establish in this city, an Institution for the *Cure of Injuries and Diseases of the* Eyes. There is at present in this County a considerable number of poor, afflicted with Diseases of the Eyes, who, unconscious that relief can be obtained, have thrown themselves on the charity of the public for support. Many who, from their infancy, have been by nature deprived of light, and thereby of the advantages of education, as well as many of a maturer age, who have remained for years in darkness, and not aware that sight can be restored to them.

The objects, then, of such an institution are, to restore the blessing of Light to many thus afflicted, and to afford assistance to those who, from neglect in the first stages of disease, pass into a state of irremediable blindness.

Institutions, for the cure of Injuries and Diseases incident to these organs, have been formed in London, and in the large towns of several counties, and the beneficial effects of such establishments will be fully recognized, on inspection of their Reports. They are found to be valuable additions to the General Infirmaries, where this particular class of diseases cannot be so nicely and tenderly treated as in institutions professedly for the purpose.

The majority of this class of diseases are capable of relief at a distance, with occasional advice and proper injunctions.

Only in severe or acute cases, or where operations are required, is it necessary to admit patients into the house; but in those daily attention is requisite.

The *out*-patients, then, comprehend a great extent of relief—they may be unlimited in number, although subject to proper regulations. The *in*-patients may be limited to six or eight.

The officers of the charity to consist of a president, governors, treasurer, committee of governors, consulting physician, two surgeons, dispensing assistant, and secretary.

Similar institutions have commenced under the superintendance of one surgeon only, and, without doubt, the business may be easily accomplished by one; but it may be more advantageous to appoint two surgeons, for the benefit of occasional consultations, and mutual assistance in operations.

We beg to offer our gratuitous assistance as surgeons to the institution.

We likewise offer the gratuitous services of one of our assistants, for dispensing medicines, during the infancy of the establishment.

If such an institution, which many gentlemen, upon our suggestion, think highly desirable, and propose to encourage, should meet with your approbation, we shall feel obliged by your informing us of it, and what donation or subscription you may be disposed to make.—We have the honour to be, sir,

Your obedient servants,

H. GILES LYFORD,

W. J. WICKHAM.

*Copy of a Letter, addressed to the Chairman of the Committee, at the County Hospital, in Winchester, on Wednesday, the 11th of July,* 1821.

Sir,—Having seen a circular letter from my junior colleagues, proposing to establish an "*Institution for the Cure of Injuries and Diseases of the* Eyes," in this city, the necessity for which they state to be grounded upon the "considerable number of poor, now in the county, afflicted with Diseases of the Eyes, who are unconscious that relief can be obtained," and because a General Hospital is not capable of affording relief to this particular class of diseases, in so eminent a degree as an institution solely appropriated to that purpose, I have felt it my duty to address some remarks upon the subject to you, and by that means to the Committee, and to the governors of the County Hospital in general.

During the ten years in which I have been surgeon to the hospital, a large share of the Ophthalmic patients have come under my care, and I have had considerable success in their cure, both by operations and constitutional treatment; and I also beg to state, that some years ago, having somewhat of a similar object in view to that which my colleagues now propose, I made inquiries of my professional brethren about the county, for cases of cataract, and other diseases of the eye, which would admit of relief by operation, but that I found by far too small a number to justify the recommendation of such a scheme to public patronage.

A very laudable zeal has no doubt prompted my colleagues to solicit the attention of the public to the present plan; but as a servant of the county, equally zealous for the promotion of every thing calculated to relieve the infirmities of the lower orders, I have no hesitation in expressing my confidence that our hospital can afford every relief to persons afflicted with diseases of the eyes in this county, and that, with very little alteration in its establishment, it might embrace every advantage which my colleagues anticipate from a distinct institution.

The alteration I mean would be the appropriation of a small room or ward, for three or four acute cases, or those requiring operations, which is the greatest number likely to be in the hospital at any one time; for it cannot be denied that the success of an operation is often dependant upon the strict quietude and particular circumspection with which the after-treatment is conducted, and therefore the seclusion of such patients from the general intercourse of the larger wards would be highly beneficial.

The number of patients annually admitted with diseases of the Eyes, and discharged cured, or greatly relieved, is a sufficient refutation of the implied aspersion that our hospital is not competent to their successful treatment, even under its present regulations; and I cannot but think that we are not warranted in imposing upon a county and a city, already abounding in charitable institutions and societies, the additional burthen of supporting a distinct infirmary for diseases of the eye, until it has been more satisfactorily proved that the County Hospital may not embrace, within itself, every advantage to be derived from such a distinct establishment.

The out-patients, which are stated to be by far the greater number expected to come under the care of the institution, cannot clearly enjoy any advantage over the out-patients of the hospital.

It is true that similar institutions have of late years been formed in London, and in three or four other large cities, as Bath, Exeter, &c. but diseases of the eyes still continue to be successfully treated in their general hospitals, and the distinct institutions have generally been under the superintendence of gentlemen who have devoted their whole attention to diseases and operations on the eyes, and the great superiority in the number of the population in those situations, has so amply furnished them with cases as to fully prove the utility of their establishment.

During the last eight years there have not been more than 160 eye cases sent to the county hospital, making an annual average of 20, of which the proportion of in-patients is 12, and of out-patients 8; and the proportion of eye-cases to all other cases admitted, is about 1 to 50. The smallness of this proportion will suffice to shew, that there could be no great difficulty or expence in providing for their occasional select accommodation, as I have above proposed.

If perchance, however, after the more particular promulgation of our willingness and competency to treat diseases of the eyes, the hospital should overflow with cases, from those uninformed districts which my colleagues mention, I shall be equally ready with them to propose a distinct establishment, and to offer my gratuitous services in its support.—I have the honor to be, sir,

Your most obedient servant,

CHARLES MAYO.

---

*Fever in Spain.*—We give the following extract of a letter from Dr. Pariset, one of the physicians who courageously undertook to visit the infected districts in Spain, as a set-off against the finely-spun vagaries of some *literary physicians*, who, never having seen the yellow fever nor the plague, venture yet to stun us with their horse-laughter at the pretended credulity of contagionists. What will the assertion of these closet-men weigh against the authority of a Pariset, who has twice been in the midst of the dreadful scenes of carnage occasioned by an infectious distemper, and now writes from the ruins of a once flourishing city?

"Yes; the disease that now devastates Barcelona is the true yellow-fever of America.

"Yes, it has been imported.

"Yes, I repeat it a thousand times, it is contagion. Let it be hoped that the facts which we have accumulated will enable us to master the partizans of a contrary system.

"Yes; the fever is a hundred times more pernicious to commerce than the most rigorous quarantine would possibly be. With only five days' good police and firmness, both Barcelona and its commerce might, humanly speaking, have been preserved, even on the avowal of the anti-contagionists of this country. But what has been the fact? They wrangled, they disputed; the scourge entered, raged, proved fatal, and nobody knew how to remedy it. Hence all labour, all industry, all prosperity, is extinct for a long time. The heart itself has partaken of this depravity. The father shuns the presence of his children, and there is an adieu to every feeling of humanity! Oh, that the administration were unceasingly vigilant, and did not tamper in this amalgamation of follies and iniquities!

"The other day it was announced to the municipal junta, that in the Calle Romada there was a shut-up house, whence there issued an offensive stench, and where an infant was heard crying time after time. The Alcaldes at length repaired to the dwelling; and, when the door was opened, there was found the body of a man disfigured greatly by the yellow fever, who appeared to have been dead about four or five days. Near the deceased lay the body of a dying woman, in a like manner disfigured by the disease; and upon her was found an infant at the breast, who, tormented by hunger, was feeding, as it cried, upon the body of the mother!

"An inn, which had fifteen travellers, lost eleven by the fever; while the adjoining house lost twenty-four out of twenty-seven.

"At Tortosa, the bishop and his family are all dead, and the episcopal palace remains empty.

"Whole congregations and fraternities in convents are dead; houses are swept of their inhabitants, and the streets are nearly deserted.

"All the secretaries of the municipality are dead.

"All the physicians, with a single exception, are also dead.

"All the apothecaries, too, excepting one gentleman, have died."

---

*More particulars of the fever.*—Dr. DALLY, Physician to the Princess of CONDE, writes from Barcelona, the 10th instant, as follows: —"The fever continues its ravages. The weather is delightful, and it is difficult to reconcile the prevalent disease with so serene a sky and an atmosphere apparently so pure. Though the town is invested, the healthy and convalescent have permission to walk in a circuit of two leagues. The number of deaths cannot be correctly ascertained. They are reckoned by some at 12, and by others at 15,000. The calls upon us to attend the sick are incessant, and increase every hour."—*Moniteur of the 15th November.*

---

*Fever in Minorca.*—The yellow fever rages in the island and in the Lazaretto, with increased violence; the mortality in consequence is very great; this must account for my not answering your last letter. We have had no packets from Spain for upwards of two months, owing to this cruel disorder. Upwards of eighty vessels of different nations are now under quarantine at this port; not one but has lost some of its crew, and a few without a single person remaining on-board. A family, within a few doors of my house, has been attacked with this dreadful malady, one of the members of which was carried off in twenty-four hours, the remainder sent to the infirmary, and the door of the infected house walled up. Several other places are surrounded by a cordon of soldiers to prevent, as much as possible, the disorder from spreading, although I much fear this will not have the desired effect. I leave you to judge what must be our feelings in such a critical situation, more particularly after having escaped the terrible plague which raged in Tunis, in the years 1785 and 1786, which was calculated to have carried off 150,000 souls, where I had also the plague in my own house.—*Extract of a letter dated Mahon, Oct.* 3, 1821.

---

*Cholera Morbus in the Island of Java.*—From accounts officially communicated it appears, that in general the Cholera Morbus, in the Island of Java was diminishing very considerably. From the Reports received from some of the Residencies it is to be observed, that many persons have been recovered by a timely use of what is called the Cholera Mixture, consisting of brandy, laudanum, and oil of peppermint; and, in consequence, thousands of the natives have eagerly applied to the local authorities for this remedy. Our papers contain the detailed reports for the 1st, 8th, 15th, 22d, and 29th June, and 21st July. The following extracts will shew the decrease of the number of deaths daily in the principal places:—

| | June 1. | June 8. | July 21. |
|---|---|---|---|
| Batavia | 35 | 12 | 5 |
| Pekalongang | 55 | 80 | 0 |
| Kandal | 98 | 80 | 9 |
| Souracacta | 17 | 30 | 10 |
| Samarang | 160 | 130 | 12 |
| Fagal | not known | 155 | not many |
| Bantan | ditto | 0 | 80 |
| Rembang | 74 | 0 | 0 |
| Patty | 59 | 137 | 4 |
| Japara | 38 | 11 | not many |
| Danisk | 0 | 280 | 24 |

The total numbers stated in the several Reports are as follow; but it must be observed in each of the Reports the number of deaths in some places are stated not to be returned, in others to be inconsiderable.

| | Deaths. |
|---|---|
| Report of June 1 | 525 |
| 8 | 1,107 |
| 15 | 958 |
| 22 | 947 |
| 29 | 1,001 |
| July 21 | 679 |

*Letter dated Hague, 10th Nov.*

*Progress of the Contagion in Spain.*—Letters were received in town on the 17th of November from Gibraltar, Barcelona, Cadiz, &c. to a late date. The accounts of the fever are as distressing as ever. The city of Barcelona, as well as Barcelonetta, continued to be in a most dreadful state. The cordon of troops between those places had received a fresh reinforcement, and the severest punishment was denounced on persons attempting to evade the restrictions imposed by the Civil Authorities and Medical Boards. The ravages of fever at Tortosa were equally extensive. Up to the 11th of the last month there had been upwards of 400 deaths in that city: numbers were lingering in a doubtful state, and the great bulk of the population had left their homes and sought refuge in the interior. We regret to find it confirmed by the present advices, that the Balearic Islands had also been visited by the fever, and were now suffering beneath that awful scourge.

---

*Cholera Morbus in Bavaria.*—By the arrival of the ship Middleburg, off Plymouth, from China and Batavia, which latter place she left on the 27th of July, letters have been received in town of the most melancholy description. They state that no less than *seventeen thousand* of the inhabitants had been carried off by the *cholera morbus.* The rice crop had failed, and in consequence the government had prohibited all exportation. Its price on-board-ship was 27, and on shore 24½. The coffee crop was very abundant, but from the dreadful malady now raging, a want of hands was experienced to pick it.

---

*New Barometer.*—Mr. Barth, of Strasburgh, has published his discovery of a *barometer,* which is to announce every change of the weather thirty hours before it happens, and to give notice of thunder storms twelve hours before they occur.

---

*Fasting for three Days in an Infant.*—On Tuesday morning, the 30th November, a child about eighteen months old was left standing at its parent's door, at Four-mile-house, near Newry, while the mother was employed in some domestic matters. In a short time the anxious parent returned for the infant, but she could not find it, after a most diligent search. The alarm being then given, the people in the neighbourhood commenced a wider and stricter search on the days of Tuesday, Wednesday, and Thursday, without success. It was then supposed that the infant had been stolen, and the distracted parents were on the eve of advertising it, but late on the evening of Thursday, the 1st inst. the child was accidentally discovered lying in the furrow of a potatoe-field, a short distance from the house, alive, and apparently little the worse for lying out without food, and scarcely any clothes, for nearly three days, and two most inclement nights.—*Newry Telegraph.*

---

*Aërolite.*—The Paris papers mention, that the stone which fell from the clouds on the 23d of June, at Javinas, in the department of Ardeche, is now exhibiting to the public. Several amateurs have made proposals for purchasing this wonderful stone, which has excited great speculation among naturalists. An English mineralogist has, we understand, offered a considerable sum for it.

---

*Organic Remains.*—THE petrified remains of a very large marine animal have been found in a quarry on the top of a hill near Bromyard. They were unfortunately removed from the quarry by the workmen, broken to pieces, and carried upon the roads in the neighbourhood. Only a part of them have, in consequence, been preserved, yet sufficient to show that the animal to which they once belonged must have been one of no common kind or size. It appears to have lain on the surface of a free-stone rock, and was covered by a thick stratum of yellowish-coloured marle. Never was the petrifying process more complete: the different pieces are literally masses of stone, only distinguishable as the parts of an animal by their exterior form.

---

*Antiepileptic Powder.*—A Dutch Baron, residing at the Hague, has of late years enjoyed no mean degree of celebrity from the invention of a powder, the composition of which is kept a secret, said to be an infallible cure for epilepsy. Not only are the Dutch, and people from other parts of the Continent, said to have experienced the wonderful effects of this nostrum; but a British nobleman of high rank, who has resided in that country, acknowledges to have been completely cured by the said powders, within the last year or two: and another nobleman, of more tender years, has since journeyed to Brussels, for the express purpose of putting himself under the care of the Baron, and of giving a trial to the antiepileptic powders. We have obtained possession of one of the powders, and intend analyzing it. The public shall have the benefit of our inquiry, should it lead to any satisfactory result. The Baron is certainly thus far different from other venders of secret medicines, that his powder is totally bereft of all those alluring *adjuvantia* to puffing—the pink and green ornaments which shine so conspicuously in the shops, even of the regular chemists of this metropolis. For the antiepileptic powder is, *tout bonnement*, put up in a piece of coarse brown paper.

---

*A new Gum-Resin.*—Mr. LAMBERT, of Lower Grosvenor-street, the well-known botanist, and one of the vice-presidents of the Linnean Society, has committed to our charge for examination, a new gum from Guiana, sent to him by a medical gentleman in the island of St. Vincent, who states, that the native Indians of that part of the new Continent use it in fumigations for coughs and other pulmonic complaints, with success. The gum is the produce of a large tree growing on the mountains of the inland parts of Guiana, and is probably a species of amyris. The gum itself is called by the natives, *akyari;* it has a resinous appearance externally, and is electric. The mass we have, and which we purpose to submit to chemical examination at our earliest convenience, is neatly enveloped in leaves of a species of musa.

*Juridical Medicine.*—A countryman and his wife, near Perth, who were in the habit of dipping the eggs intended for market in a solution of vitriol, to whiten them, and give them a fresh appearance, had a dispute a few days since, when the husband attempted to throw a bottle of vitriol at his wife. She intercepted it with her hands, by which the bottle was broken, and the contents thrown back in the face of the husband, who has been blind ever since, and will never, in all probability, recover the use of his eyes.—*Glasgow Chronicle.*

---

*Infanticide.*—Recently an inquisition was taken at the Unicorn, in Covent-Garden, before Thomas Higgs, esq. on the body of a fine male child, who was found murdered in Covent-Garden market. Henry Hunt deposed that he lived with Mrs. Grimley, herborist, in the market. On Wednesday evening a man, who was a stranger, came to the shop, saying, there was an infant lying dead on the ground at the back of Mr. Cook's room, in the market, and requested him to get a light; he did so and went to the spot, where the child was discovered wrapped up in a cloth, suffused with blood. It appeared to have been dead about three days. Information was given to the parish officers, and the body was conveyed to the watch-house. Dr. Morris, of Chandos-street, described the situation of the child. It had received six blows; three were on the temple and three on the breast, and had, in his opinion, been inflicted by a red-hot poker, which caused its death. He had no doubt it was born alive, and was a remarkably full grown child.

The Jury, without hesitation, returned a verdict of wilful murder against some person or persons unknown.

---

A coroner's inquest was taken at Old Hurst, Hunts, on a cottager's child two years of age, who had been accidentally killed by a pocket-knife, which it held in its hand. The child was running across the sitting-room, when a projecting brick caught its foot, and in falling, the knife entered its throat under the auricular glands. —*Lincoln Gazette.*

---

Dr. Bancroft.—Lately died at Margate, aged 76, Dr. Edward Bartholomew Bancroft. This gentleman was bred to physic, and being admitted to his degrees, was, when young, physician to the army. In this capacity he resided for some time in the West Indies, and was afterwards a member of the College of Physicians. He was the author of several useful works, among which is "An Essay on the Natural History of Guiana, in South America," 8vo. 1769. He did not confine himself to books on his own profession; but, in 1770, he published "The History of Charles Wentworth," a novel, 3 vols. In 1794, "Experimental Researches concerning the Philosophy of Permanent Colours, and the best way of producing them by Dyeing, Calico-printing, &c." of which an enlarged edition was published in 1813, and it is a work held in high estimation by manufacturers and experimental philosophers; also "An Essay on the Yellow-Fever." Dr. B. entered into the dispute respecting the military inquiry, and published a Letter to the Commissioners on their Fifth Report, and a Refutation of various Misrepresentations, published by Drs. Macgregor and Jackson.

---

Mr. Wilson.—Quam brevis vita!—We were far, very far, from thinking, while employed in paying a just tribute of praise to the professional qualification of this gentleman in our last Number, that ere our pages were yet dry, we should have to record the heavy loss which his family, his friends, and the public, have sustained by the sudden and awful visitation which has called an excellent man from hence, "to another and a better world."

---

*Another Expedition into the Interior of Africa.*—Captain Denham, who served with the Duke of Wellington's army in the Peninsula and at Waterloo, arrived here on the 20th, in the Walsingham packet, on his way to Tripoli, where he will take charge of an expedition, which has been some time in agitation, for exploring the interior of Africa; he will be accompanied by a Dr. Oudeny and Lieut. Clapperton, of the navy; the former, a member of the University of Edinburgh and a skilful and intelligent naturalist, will find a wide and interesting field for the exercise of his talents by a residence at Bornou, where he will remain as vice consul, and by that means a *point d'appui* will be established nearly 1,000 miles from Tripoli, where the expedition may rest during their subsequent operations, to the eastward and southward of that place. A naval carpenter, from the Dock-yard at Malta, will also accompany these gentlemen in their interesting and hazardous undertaking. Captain Denham will have the local rank of major on the continent of Africa.—*Extract of a Letter from Gibralter, dated October* 25.

---

*The Phosphorescence of the Lampyris Noctiluca and Splendidula.*—In a curious paper on the phosphorescence of the lampyrus noctiluca and splendidula, Mr. Macaire has drawn the following conclusions from numerous observations:—1. A certain degree of heat is necessary to the voluntary phosphorescence of these animals. 2. Their phosphorescence is excited by a degree of heat superior to the first, and is irrecoverably destroyed by a higher temperature. 3. All bodies capable of coagulating albumen take away, from phosphorising matter, its power of phosphorescence. 4. The phosphorescence cannot take place but in a gas which contains oxygen. 5. It is excited by the galvanic pile, but no effect is produced upon it by electricity. 6. The phosphorescent matter is composed principally of albumen.—*Bibliotheque Universelle, May* 1821.

---

*Account of the Leech of Ceylon.*—This animal is seldom more than half an inch long, and is nearly semi-transparent. It is very active, and is said occasionally to spring. Its powers of contraction and extension are very great. It is like a fine chord when fully extended; and its point is so sharp that it easily makes its way through very small openings. It is supposed to have an acute sense of smell; for, no sooner does a person stop where leeches abound, than they appear to crowd eagerly to the spot from all quarters. "Those who have had no experience of these animals," says Dr. Davy; "of their immense numbers in their favourite haunts,—of their activity, keen appetite, and love of blood,—can have no idea of the kind and extent of annoyance they are to travellers in the interior, of which they may be truly said to be the plague. In rainy weather, it is almost shocking to see the legs of men on a long march, thickly beset with them, gorged with blood, and the blood trickling down in streams. In attempting to keep them off, they crowd to the attack, and fasten on quicker than they can be removed. I do not exaggerate when I say that I have occasionally seen at least fifty on a person at a time. Their bites are apt to fester and become sores, and frequently degenerate into extensive ulcers, which, in too many instances, have occasioned the loss of a limb, and even of life."—*Dr. Davy's Account of the Interior of Ceylon.*

---

*Propagation of Sound in Elastic Fluids.*—Mr. Van Rees has given the results of his experiments on the propagation of sound on elastic fluids, made with great care, and under the auspices of MM. Frameyer and Moll. The following are some of the results:—

| | Velocity 10° of Centig. theorem. |
|---|---|
| Hydrogen . . . . . . . . . | 1233.3 metres. |
| Ammonia . . . . . . . . . | 432 |
| Vapour of water, temp. 54° cent. | 422.6 |
| Carbonic oxide . . . . . . . | 341.1 |
| Azote . . . . . . . . . . . . | 339.0 |
| Carburetted hydrogen . . . . | 377.4 |
| Oxygen . . . . . . . . . . . | 317.7 |
| Deutoxide of azote . . . . . | 317.4 |
| Sulphuretted hydrogen . . . . | 305.7 |
| Hydrochloric acid . . . . . | 298.8 |
| Carbonic acid . . . . . . . . | 270.7 |
| Protoxide of azote . . . . . | 270.6 |
| Vapour of alcohol . . . . . . | 262.7 |
| Sulphurous acid . . . . . . | 229.2 |

*Journal de Physique, Jan.* 1821.

---

*Partial Palsy removed by Lightning.*—Mr. Olmsted, professor of chemistry in the college of North Carolina, has published an account of the removal of a paralytic affection by a stroke of lightning. Mr. Samuel Leffers, of Carteret county, North Carolina, had been affected with a paralytic affection in his face, which had settled chiefly in the eye. When he was walking in his house during a thunder-storm, he was struck down by lightning. After lying senseless fifteen or twenty minutes, he recovered so far as to be sensible of his situation. He recovered the use of his senses and of his limbs by degrees, during the remainder of the day and night; and he felt so well the next day, that he was inclined to give to a distant friend an account of what had happened. He was able to write a long letter without the use of glasses. Since that time he never felt a symptom of the paralytic disorder; and he concluded that it had been effectually cured by the shock: he thought, however, that the same cause which restored his sight impaired his hearing.—*American Journal of Sciences*, vol. iii.

---

*Plant soluble in Water.*—THE plant called *nostoch communis*, which is found in the South of France, in the form of a green and membranaceous envelope, filled with a species of jelly, containing a number of elongated filaments, has the remarkable property of dissolving in water. It always disappears when the rain has ceased, leaving only a small dry membrane, apparently inorganized, which resumes its original form by being wetted. A curious paper on this plant, and on the different names it has received, is published by Mr. VALLOT.—*Journal de Physic, Mars* 1821.

---

*Effects of Copper on Vegetation.*—SOME time since, says Mr. PHILLIPS, having accidentally spilt some solution and oxide of copper near the root of a young poplar tree, in a short time the tree began to droop; the leaves on the lower branches dying first, and eventually those on the upper ones. On cutting a branch from the tree, he observed that the knife was covered with copper to the whole breadth of the branch, showing that the copper had been absorbed, and had undoubtedly proved fatal to the life of the tree.—*Ann. Phil.*

---

*Lactometer.*—MR. DAVY, the professor of chemistry to the Royal Cork Institution, has published some experiments made with a view to the detection and prevention of frauds in the sale of skimmed milk. He has invented a simple instrument for effecting that purpose, of which there is a good plate in the last Number of the Philosophical Magazine. When it is considered to what extent skimmed milk is used in Ireland, particularly in that part of it in which the professor resides, where it forms an indispensable part of the subsistence of the lower orders, the advantages of such an invention will be readily admitted. We shall give the paper itself in one of our next Numbers.

---

*Effects of Metallic Salts on Blue Vegetable Colours.*—IN a series of experiments, lately made on vegetable colours, Mr. J. MURRAY has discovered the remarkable fact, that subacetate of lead, nitrate and sulphate of copper, nitromuriate of platinum, nitromuriate of gold, &c. turned syrup of violets, tincture of cabbage, columbine, blue hyacinth, &c. *green*; and that, when these colours are even reddened by acetic or citric or carbonic acid, &c. the metallic solutions *restore the original blue* colour. Boracic acid reddens the yellow colour obtained from *reseda lutea*, and so do the metallic solutions. It seems evident, therefore, that we have yet to learn the invariable characteristics of alkalies and acids.

*Analysis of Mineral Waters.*—In the last volume of the Memoirs of the Literary and Philosophical Society of Manchester, there is a valuable paper by John Dalton, containing some remarks tending to facilitate the analysis of spring and mineral waters, which we recommend strongly to those who are anxious to acquire that degree of accuracy in this sort of delicate chemical operations, which is not to be obtained by the perusal of certain other recent essays on the same subject, coming from an imposing quarter.

---

*Abstract of Fifty Operations for Hernia.*—These fifty cases came under the observation of Dr. Massalien, during a practice of thirty years, previously to the year 1810. Of these cases there were—

| | |
|---|---|
| 29 in males and .............. | 21 in females. |
| Of which there were 25 inguinal and 4 femoral. | Of which there were 5 inguinal, and 16 femoral. |
| 11 men and .................. | 9 women died. |
| 19 inguinal and 3 femoral were on the right side; 5 inguinal and 1 femoral on the left. | 5 inguinal were on the right side; 9 femoral on the right, and 7 on the left. |

—*Zeitschrift für Natur und Heilkunde, Dresden.*

---

*Transposition of the Abdominal Viscera.*—An instance of this kind has presented itself to the observation of Dr. Campbell, of Edinburgh, who has published a detailed account of it in the 69th Number of the Edinburgh Medical Journal. In the cavity of the abdomen there were found only the liver and gall-bladder, the cardiac portion of the stomach, the termination of the colon, and the kidneys, covered with the peritoneum. The rest of the stomach, the small intestines, and the remainder of the colon, with the spleen, pancreas, and great omentum, were found in the left thoracic cavity. It appears, from a note by Dr. Campbell, that the *sapientes* (good folks !) of the northern Alma Mater called in question the occurrence of this case, as well as the probability of the child continuing to live under such unusual circumstances; and that he was, in consequence, obliged to refresh their memory with the relation of several instances of visceral transposition, taken from British and foreign authors, in support of his own accuracy. Dr. Campbell might have mentioned among them the very striking case recorded by the Editor of this Journal, in one of his letters on the State of Medical Science in France, inserted in the 7th volume of the Medical Repository; where he would have found that a female, who died at the age of fifty years, exhibited, on dissection, a complete reversion of the principal thoracic and abdominal viscera. But we have had occasion to mark the studied manner in which the good people of Edinburgh connected with the medical press avoid mentioning even the bare name of the Editor in question; for they even forgot to acknowledge the receipt of his books, sent them as presents,—an honour which they have not denied to Mr. Callow's General Catalogue of Second-hand Books.

*Spina Bifida.*—Through the kindness of Mr. Jukes, the very respectable practitioner of Peter-street, Westminster, we have been allowed to inspect one of the most remarkable cases of spina bifida (if, indeed, it be not the only one of the kind on record,) that we had ever seen, heard, or read of. The subject is a young woman, now aged nineteen years, who was born with a small vesicular swelling at the lower extremity of the dorsal spine, which swelling has kept increasing every year, so as to have now reached a size considerably larger than that of an ordinary man's head. It contains a transparent fluid, which occasionally oozes, in very small quantities, through the integuments by which the tumor is formed. This young woman has enjoyed good health ever since, and menstruates through an open sore in the fleshy part of the right thigh. We entertain no doubt that Mr. Jukes, who has attended the patient for a long time, would procure, willingly, to any respectable member of the profession who should wish to inspect this pathological phenomenon, the means of doing so, on a slight remuneration being made to the parents of the patient, who are in needy circumstances. Mr. Jukes has led us to hope that we shall soon be enabled to lay before our readers a more circumstantial account of this case, with a drawing of the part, from himself.

---

*Journal of Experimental Philosophy.*—The fourth Number of Dr. Magendie's excellent Journal has been laying before us for some time. It contains, 1, a continuation of Dr. Roulin's Inquiry into the Nature of the Movements and Attitudes of Man. 2. Observations on a Rupture of the Stomach in the Horse, by Mr. Dupuy. 3. On the Organs which tighten or relax the Membrane of the Tympanum and the Ossiculi of the Auditory Apparatus, in Man and mammiferous Animals, by Dr. Magendie. 4. Note on the *Pian,* a pustulous Eruption of the Skin common in the West Indies, by Rochoux. 5. Considerations on the Organic Derangement called *Melanose,* by Breschet. 6. Anatomical Examination of a Dog, having only one Eye and no Mouth, by Magendie. 7. On the Use of Electricity in Concussions of the Brain, by Gondret. 8. An Abstract of Mr. Charles Bell's System of Nerves. 9. Chemical Analysis of the Guttural Ganglions of the Trisplanchnic Nerve in the Horse, by Lassaigne. 10. Case of *pernicious* Intermittent Fever cured with a small Dose of the Sulphate of Quinine, by Magendie. This Number is illustrated with four plates: the first of which represents the comparative anatomical structure of the ossiculi in the ear of twelve different mammiferous animals; the second, a variety of views of [illegible] same organs in man; the third, some representations of the monoculous and astomic dog described by Dr. Magendie; and the fourth, several diagramatic illustrations of the paper on muscular motion.

---

*Medical Instruction.*—The French government has determined to establish a School of Medicine at Lyons. Besides the usual course of Lectures delivered by the physicians and surgeons of hospitals, there will be a class for clinical medicine, a second for internal pathology, and a third for therapeutics and materia medica. The professors' chairs will be filled by persons who shall have undergone certain probatory examinations, in the presence of the members of the administration of hospitals, assisted by a jury of medical practitioners.—*Tablettes Historiques, August.*

---

*Phthisis Laryngæa.*—Dr. Siemerling, of Berlin, who cured his own wife and several others of the above complaint, has given publicity, in the Prussian Gazettes, to his mode of treatment, which consists in desiring the patients to eat, every morning fasting, the *roe* of a large salt Dutch herring!!—*Tablettes Historiques, August.*

---

*Medical Promotion.*—We have been informed that Dr. Magendie has been appointed, by the Faculty of Medicine of Paris, to fill the chair vacant by the death of Corvisart. The unsuccessful candidate on the occasion was Professor Chaussier.—*Paris Letter, Nov.* 20.

---

*Public Expression of Gratitude to a Physician.*—The medical students at the university of Paris have entered into a resolution of erecting a public monument to the memory of Dr. Mazet, who perished in Spain from the effects of the contagious fever, the nature of which he had gone to investigate in company with Drs. Bally, François, and Pariset. Dr. Bally, whose death was also reported, is, we are happy to say, fast recovering from the same disorder.—*Paris Letter,* 21*st November.*

---

*Death of Dr. Rigby.*—Lately died, at his house in Giles's, Edward Rigby, Esq. m.d. whose talents, character, and honours, claim from us a record, which, in common with so large a circle of friends, connexions, and admirers, as it falls to the lot of very few men to possess, we lament was not postponed to a more distant date; for it may be said, in the language of Burke, that "he was a public creature,—he had in himself a salient, living spring of generous and manly action;" and the longer (as it seems to human apprehension,) life had been indulged to him, the greater would have been the benefits that life would have enabled him to diffuse amongst his fellow creatures,—for *usefulness* was the motive, path, and end of his existence.

---

*Galvanic experiments on a Criminal.*—The body of George Thom, who was executed here last week, having, agreeably to his sentence, been given for dissection to Drs. Skene and Ewing, was subjected to a series of Galvanic experiments, of which, with their results, we subjoin the following brief account:—The body was brought into the dissecting room about an hour after suspension, and still retained nearly its natural heat. The upper part of the spinal cord and the sciatic nerve were immediately laid bare, and a Galvanic arc was then established, by applying the positive wire to the spine, and the negative to the sciatic nerve, when a general convulsive starting of the body was produced. Another communication was then made between the spine and the ulnar nerve, and considerable contractions took place in the arm and fore-arm. When the circle was formed with the spine and radical nerve, both at the elbow and wrist successively, powerful contractions of the muscles of the whole arm and hand were produced. The hand was closed with such violence as to resist the exertions of one of the assistants to keep it open. When a connection was established between the radical nerve and the supra and infra orbital nerves, strong contractions of the muscles of the brow, face, and mouth were produced, so as to affect the under jaw, and to distort the countenance in a very singular manner. The eye-lids were strongly contracted; and when the wire was applied directly to the ball of the eye, the iris contracted and dilated very sensibly. A galvanic circle being formed, first between the par vagum and diaphragm, and then between the muscle and the great sympathetic, little obvious effect was produced. After applying galvanism directly to the nerves above mentioned, the skin of the face was moistened with water, and upon running the wire over the different parts of it, similar effects were produced in the muscles of the face, as by direct communication made with the nerves. The tongue also moved in all directions, by touching the surface with the galvanic wire. The whole experiments were performed in about an hour and a quarter, when the heat of the body was considerably diminished. A powerful galvanic apparatus (consisting of about 300 pair of plates) was used; but, from not being insulated, a considerable quantity of the galvanism escaped, so that every metallic substance about the table was highly charged. It is not improbable that a more particular and scientific account of these experiments may yet be given to the public; but, in the mean time, the accuracy of the above general statement may be relied on.—*Aberdeen Journal.*

---

Such was the dread of the fever which has been raging at Barcelona, that almost all the principal members of the faculty, contagionists and noncontagionists, when their aid was most wanted, left the town in the most indecent haste. In consequence of such unprecedented conduct on the part of the medical practitioners, the first constitutional alcalde, with the advice and concurrence of the *Junta de Sanidad*, has enjoined the fugitives to return to their duty, on pain of having their property confiscated, and their diploma cancelled, in the event of noncompliance to this order. Many of the noncontagionists, who, never having seen the yellow fever, except in print, can sit coolly down and indite letters and essays on its perfectly harmless nature, would perhaps have imitated the Barcelona doctors, had they been placed in a similar perilous situation.—*Decadas Medico-Quirurgicas,* Tom. iv. No. 111.)

---

*Medical Bibliography.*—Among the very numerous works we have received from abroad for review, we may notice the following; many of which will be analyzed in due course:

*From France.*

1. Recherches sur l'Inflammation de l'Arachnoïde Cerebrale et Spinale. Par Mess. Parent Duchatelet et L. Martinet. Paris, 1821. 1 vol. 8vo. pp. xxiv. 608.

2. Recueil de Memoires de Chirurgie. Par le Baron Larrey. Paris, 1821. pp. xvi. 320, plates.

3. Pratique des Accouchemens; ou Memoires et Observations choisies sur les faits les plus Importants de l'Art. Par Madame Lachapelle. Paris, 1821. 1 vol. 8vo. pp. x. 524; Tables.

4. Traitè des Maladies de l'Oreille et de l'Audition. Par J. M. G. Itard. Paris, 1821. 2 vol. 8vo. pp. 396, 522; plates.

5. Essai sur les Irritations intermittentes, &c. Par C. J. Mougellaz, D.M. Paris, 1821. 2 vol. pp. x. 464, 394.

6. Dictionnaire de Medecine. Par Mess. Adelon, Beclar, Biet, Breschet, Cloquet, &c. &c. 1st vol. 8vo. pp. 563. Lett. A, ALI. 2d vol. pp. 568 to ABG. Paris, 1821.

7. Nouveau Dictionnaire de Medecine, Chirurgie, Pharmacie, Physique, Chimie, Histoire Naturelle, en 2 volumes. Vol. 1. 8vo. pp. 829. Paris, 1821.

8. Rapports et Consultations de Medecine Legale recueillies et publièes. Par J. Ristelhueber. Paris, 1821. 8vo. pp. 182.

*From Italy.*

9. Prospetto Clinico dell' Anno Scolastico, 1819—1820. Del Cavalier Luigi Brera. 8vo. Padova, 1821. pp. 122; Tables.

10. Sopra un Nuovo Antidoto pel Sublimato Corrosivo. Del Dr. G. Taddei. 8vo. Florence, 1820.

11. Fondamenti di Patalogia Analitica. Di Maurizio Buffalini. 2 vol. 8vo. Pavia, 1820.

12. Discorsi due sulla Medicina. Del Signor A. Chiappa. 8vo. 1821.

*From Germany.*

13. De Aure et Auditu Hominis et Animalium. Part I. De aure animalium Aquatilium Auctore. E. H. Webero. Cum tabulis æneis 4to. Lipsice, 1820.

14. Deutsches Archiv fur die Physiologie. Sechster Band, Drittes Heft. By J. F. Meckel. Berlin, 1821. 8vo.

14. Allgemeine Medizinische Annalen, &c. for July, 1821. 4to. Leipzig.

16. Beobactungen im Gebiete der Ausubenden Heilkunde. Von Johann Heinrich Kopp. Frankfort, 1821.

*From America.*

17. The Pharmacopœia of the United States of America. 1820. 8vo. pp. 272. Boston; (at the Custom-house.)

18. Account of the Yellow or malignant Fever, as it occurred in the city of Philadelphia in 1820. By J. Jackson, M.D.

19. The Philadelphia Medical Journal for May 1821.

*From India.*

20. An Essay on the Epidemic Cholera of India. By Reginald Orton, assistant-surgeon to the 34th regiment of foot. 2 vols. Vol. 1. pp. 421. Madras, 1820.

21. Remarks upon the Morbus Oryzeus (Cholera Morbus of India). By Robert Tytler, M.D. M.A.S. 2 Parts. pp. 147, 152. Calcutta, 1820.

### METEOROLOGICAL JOURNAL.

By Messrs. WILLIAM HARRIS and Co. 50, Holborn, London.

*From October* 20 *to November* 19, *inclusive.*

| Day of Month. | Moon. | Rain gauge | THERM. | | | BAROM. | | DeLuc's Hygro. | | WIND. | | ATMOSPHERIC VARIATION. | | |
|---|---|---|---|---|---|---|---|---|---|---|---|---|---|---|
| Oct. | | | | | | | | | | | | | | |
| 20 | | .09 | 48 | 52 | 44 | 29.21 | 29.10 | 57 | 55 | SW | SW | Cloudy | Rain | Cloud. |
| 21 | | .17 | 46 | 49 | 44 | 29.21 | 29.18 | 54 | 56 | W | SE | Fine | | Rain |
| 22 | | | 47 | 52 | 46 | 29.20 | 29.37 | 57 | 57 | SW | SW | Cloudy | Fine | |
| 23 | | .11 | 49 | 53 | 44 | 29.34 | 29.38 | 57 | 60 | SSW | W | Cloudy | Rain | Fine |
| 24 | | .18 | 45 | 50 | 46 | 29.46 | 29.83 | 57 | 55 | W | W | Foggy | Rain | Sho'ry |
| 25 | | .10 | 49 | 52 | 45 | 29.90 | 30.00 | 53 | 55 | W | WNW | Foggy | Rain | Cloud. |
| 26 | ● | | 49 | 55 | 45 | 30.01 | 30.12 | 55 | 60 | NW | W | Foggy | Cloud. | |
| 27 | | .24 | 50 | 53 | 43 | 30.17 | 30.23 | 55 | 57 | WSW | SW | Rain | | |
| 28 | | .03 | 48 | 57 | 43 | 30.27 | 30.30 | 55 | 57 | SW | SSW | Fine | | Foggy |
| 29 | | | 47 | 55 | 46 | 30.25 | 30.19 | 56 | 59 | SE | SE | Foggy | | |
| 30 | | | 49 | 58 | 47 | 30.12 | 30.05 | 60 | 57 | SW | SSW | Foggy | Fine | Foggy |
| 31 | | .04 | 48 | 60 | 46 | 30.00 | 30.00 | 59 | 56 | SW | SSW | Foggy | Cloud. | Fine |
| Nov | | | | | | | | | | | | | | |
| 1 | | | 50 | 64 | 47 | 30.00 | 29.96 | 60 | 55 | SW | SW | Foggy | Fine | Cloud. |
| 2 | | | 50 | 63 | 46 | 29.90 | 29.85 | 59 | 57 | WSW | W | Fine | | Cloud. |
| 3 | ☾ | .10 | 51 | 60 | 43 | 29.61 | 29.50 | 55 | 50 | SSW | WSW | Cloudy | Fine | Rain |
| 4 | | .15 | 49 | 60 | 40 | 29.33 | 29.51 | 45 | 49 | W | W | Fine | Rain | Fine |
| 5 | | | 45 | 53 | 37 | 30.10 | 30.25 | 50 | 49 | NW | NW | Cloudy | Fine | |
| 6 | | | 41 | 47 | 39 | 30.29 | 30.33 | 50 | 53 | W | W | Foggy | Fine | Foggy |
| 7 | | | 45 | 52 | 37 | 30.28 | 30.21 | 50 | 51 | SE | SSE | Foggy | Fine | Cloud. |
| 8 | | | 39 | 49 | 38 | 30.19 | 30.16 | 50 | 50 | SE | SE | Cloudy | Fine | |
| 9 | ○ | | 41 | 48 | 43 | 30.19 | 30.15 | 55 | 54 | W | W | Cloudy | Fine | Cloud. |
| 10 | | | 45 | 53 | 50 | 30.12 | 30.18 | 60 | 63 | SE | SW | Foggy | | |
| 11 | | .23 | 53 | 59 | 48 | 29.92 | 29.76 | 65 | 65 | SSW | SW | Cloudy | Rain | |
| 12 | | .09 | 52 | 58 | 50 | 29.87 | 30.04 | 63 | 60 | SE | W | Cloudy | Rain | Cloud. |
| 13 | | .18 | 53 | 60 | 50 | 30.00 | 29.72 | 65 | 67 | S var. | SW | Foggy | Cloud. | Rain |
| 14 | | .13 | 53 | 59 | 51 | 29.77 | 29.81 | 63 | 65 | SW | SSW | Foggy | Cloud. | Rain |
| 15 | | .18 | 55 | 59 | 50 | 29.73 | 29.53 | 64 | 65 | SW | WSW | Cloudy | Sho'ry | Rain |
| 16 | ☽ | .40 | 58 | 60 | 50 | 29.49 | 29.43 | 67 | 66 | SW | SW | Cloudy | Rain | |
| 17 | | .70 | 53 | 58 | 52 | 29.61 | 29.68 | 57 | 66 | WSW | S | Fine | Cloud. | Rain |
| 18 | | | 50 | 49 | 50 | 29.93 | 30.13 | 58 | 58 | NNE | ESE | Fine | | |
| 19 | | .60 | 50 | 52 | 44 | 30.01 | 30.00 | 59 | 55 | S | WSW | Foggy | Rain | Fine |

The quantity of rain fallen in the month of October, was 2 inches and 23-100ths.

